# Reproductive Biology and Early Life History of the Apodid Sea Cucumber *Chiridota laevis*

**DOI:** 10.3390/biology14111471

**Published:** 2025-10-22

**Authors:** Sara Jobson, Jean-François Hamel, Annie Mercier

**Affiliations:** 1 Department of Ocean Sciences, Memorial University, St. John’s, NL A1C 5S7, Canada; 2 Society for the Exploration and Valuing of the Environment (SEVE), Portugal Cove-St. Philip’s, NL A1M 2B7, Canada; jfhamel.seve@gmail.com

**Keywords:** spawning, development, oocyte, embryo, hermaphroditism, holothuroid, echinodermata

## Abstract

**Simple Summary:**

Sea cucumbers are keystone species of marine ecosystems around the world that demonstrate diverse life history strategies. *Chiridota laevis* is a burrowing species found in temperate to cold waters of the Arctic, North Atlantic, and North Pacific Oceans. The widespread distribution of this species underscores the value of studying the currently unknown fundamentals of its reproduction and development. In the present study, adults displayed distinct male or female sexual cells (sperm or eggs) for most of the year before undergoing a seasonal sex change in the fall months. Spawning and fertilization occurred in late winter, coinciding with the coldest water temperatures. The sticky eggs of *C. laevis* sank immediately after being released and formed a mat on the muddy sediment. The embryos were ~350 μm in diameter and did not undergo a feeding larval stage, indicating that yolk reserves sustained the developing offspring. The development of *C. laevis* was relatively long, with individuals hatching at 7 weeks and beginning to feed at 10 weeks. The measured growth rate suggests that the species may take up to 7 years to reach the adult size. Assessing the life history strategy of this understudied species helps us understand its unique evolutionary position and ecological role.

**Abstract:**

The apodid sea cucumber *Chiridota laevis* has been a documented member of endobenthic marine communities in northern waters for over a century and the rare studies available on its biology identify it as distinctive species and promising model for research. The present study sought to elucidate fundamental aspects of its life history that remained unresolved. Adults were determined to be protandric, with individuals primarily demonstrating solely male or female gametes from winter (close to spawning) to the spring and summer months before undergoing a sex change in the fall months. Additionally, gametes of both sexes reached maturity synchronously in late winter (February to March). In mesocosms, free spawning occurred in February, as the temperature reached ~2.0 °C. The negatively buoyant eggs were encased in a sticky casing and fell to the sediment where they adhered to each other to form a mat on the muddy substratum. The realized fecundity was ~15,000 offspring. Development was lecithotrophic, demersal, and abbreviated, characterized by the absence of a pelagic larval stage. Embryos reached the gastrula stage after about 7 days post fertilization; the calcareous ring appeared at 6 weeks, and juveniles hatched from the sticky casing at 7 weeks, immediately becoming endobenthic. The size of late embryos and juveniles remained similar (~350 μm) until they began actively feeding at about 10 weeks of age. Feeding juveniles more than doubled in size in the first week (740 μm), reached 3.5 mm by year one, and measured up to 11 mm by year two. This growth rate suggests that it may take this species up to 7 years to reach adult size at ~24 mm contracted length.

## 1. Introduction

The apodids make up ~17% of holothuroid echinoderms [1,2] and yet there is limited information on fundamental aspects of their biology (e.g., reproduction, development, feeding, habitat engineering). This unique order of holothuroid was originally defined based on the morphology of their tentacles (pinnate, digitae, and peltatodigitae, [1,3]), body shape (vermiform, [4,5]), calcareous ring (size and shape, [6,7]), and their lack of ambulacral podia (i.e., tube feet, [8]). More recently, however, their ossicle shape (wheel, rod, or anchor, [9]) has been used as a means of refining the taxonomic position of species [10]. Apodids are generally considered small sea cucumbers (lower size limit < 28 mm, [11]); however, some species can grow up to 300 cm in length [12].

While dioecy, or gonochorism (Supplementary Table S1), has been considered the most common expression of sexuality in holothuroids [12,13], apodids demonstrate various forms of sexuality, including dioecy (e.g., *Synapta maculata*, [14]), hermaphroditism (e.g., *Rhabdomolgus ruber*, [12]), and protandry (e.g., *Polycheira rufescens*, [15]). Hermaphroditism has been observed in *Leptosynapta inhaerens*, *L. minuta*, *L. tenuis*, *R. ruber*, *P. rufescens*, and *S. vivipara* [14,16,17]. Sequential hermaphroditism or sex change has been documented in three temperate species (i.e., *L. clarki*, *Patinapta ooplax,* and *P. rufescens*, [15,18,19,20]). More precisely, the apodid species *L. clarki* was studied off the west coast of Canada and found to undergo a sex change during the coldest months (January–February), a few weeks after their annual spawning period of November–December [17,21]. In this species, all juveniles are male with some individuals undergoing a sex change following reproductive maturity (between 20 and 50 mm in length) resulting in an adult population with a 1:1 ratio of males and females [21]. Two apodid species sampled off the coast of Japan (*P. ooplax* and *P. rufescens*) demonstrated reciprocal sex changes (male to female to male) occurring within two-week windows between multiple spawning events. This reproductive sequence (spawning followed by sex change) occurred during the warmest months (July–October) and immediately following new and full moons [15,19,20].

Like many holothuroids, most apodids possess ramified or branched gonad tubules [15,22] grouped in either a single [14] or double tuft [23]. Irrespective of the number of tufts, tubules are often described as stemming from a gonoduct situated in the dorsal mesentery [24,25,26,27,28]. While detailed morphometrics of apodid gonad tubules are not commonly discussed, Frick [29] noted that mature *S. hydriformis* demonstrated a gonad with two short tubules that were maximally 25% the overall length of the individual. Mature males of *L. clarki* were noted to hold a pair of tufted gonad occupying most of the perivisceral coelom [26]. Comparatively, females of *L. clarki* also exhibit a pair of tufted gonads; however, ovaries in this species are generally shorter and wider than the testis [17].

Apodids exhibit either annual, biennial, or monthly gamete synthesis [14,15,20,22] primarily documented as a response to new and full lunar phases (e.g., *P. ooplax* from southern Japan, [20]), temperature increases (e.g., *P. rufescens* from southern Japan, [15]), and nutrient availability from phytoplankton blooms (e.g., *P. rufescens*, studied as *P. fusca*, from southern Taiwan, [14]). Most apodid species studied so far have shown gamete development during warm seasons (e.g., summer and fall). For example, in *P. ooplax* and *P. rufescens*, gametogenesis occurs primarily during the summer months, with gamete growth happening in the two months prior to spawning in late summer to early fall (i.e., July–October, [15,20]). Subsequently, the “cleaning” of the gonad through the resorption of residual gametes (often referred to as the recovery phase, [30]) occurs in the 2 mo following spawning [15,20]. In contrast, *Synaptula hydriformis* undergoes asynchronous gamete synthesis throughout the year allowing individuals to brood several cohorts of juveniles within a short time frame [29]. Due to this asynchronous development, the gonad of *S. hydriformis* is generally considered to be in a constant state of gamete growth, failing to undergo the recovery and resorption phase seen in many other apodids [31]. In *L. clarki*, gamete synthesis is reportedly slower, taking place across 7 mo (i.e., roughly from May to December, [17]).

Until recently, all holothuroids including apodids were believed to demonstrate conventional ovulation strategies, whereby final meiotic maturation (including germinal vesicle breakdown and the release of polar bodies) occurs during transit along the gonoduct at the time of spawning or upon contact with seawater. However, a recent study on *Chiridota laevis* outlined an unusual pattern whereby final meiotic maturation occurs inside the gonad tubule days to weeks prior to spawning [32]. Until that study, echinoids were believed to be the only class within the animal kingdom to demonstrate this unique reproductive strategy [33,34].

Apodids have been classified as either broadcast spawners [20] or brooders (viviparous or oviparous, [28,35]). Broadcast spawners like *P. rufescens* (studied as *P. fusca*, [19]) release gametes into the water column to be externally fertilized, whereas brooding species like *R. ruber* rely on pseudocopulation [11,12]. *Synaptula hydriformis* represents a special case, whereby oocytes are released into the perivisceral coelom where they are self-fertilized and subsequently brooded [36].

Apodids have been documented to produce propagules that rely on all three nutritional modes (planktotrophy, lecithotrophy, and matrotrophy). Similarly to what is seen in other holothuroids, planktotrophy (e.g., *Chiridota gigas*, [37,38]) and lecithotrophy (e.g., *L. minuta*, [39]) are the most common modes in Apodida. Matrotrophy has been documented in two species of apodids (i.e., *L. clarki* and *S. hydriformis*, [18,36]). This rarer nutritional mode involves parental supplementation of extra-vitelline nutrition to the young during brooding.

Relatively little is known about the embryonic development and/or juvenile growth of apodid sea cucumbers. Most knowledge comes from the study of the internal brooders, including *L. clarki* [17,18,21,28,40,41,42], *C. rotifera* [35], and *S. hydriformis* [29,36,43,44], and of the broadcast spawners *R. ruber* [27] and *C. gigas* [38]. Of the internal brooders, *L. clarki* demonstrates abbreviated development, with embryos becoming pentactulae within 2 weeks and the juveniles remaining in the ovary for up to 6 mo [18,42]. After their release, juveniles experience rapid growth, reaching adult size in 6 mo and becoming mature adult males by the end of their 1st year [18]. Two other brooders, *C. rotifera* and *S. hydriformis*, develop from a gastrula to early pentactula [35,36,45], supporting the idea that they are also abbreviated (nearly direct) developers. While brooded pentactulae have been described in both species [35,45], there is little to no information on the release of juveniles from the adults in these species and the time to reach sexual maturity is unknown. Similarly limited data exist about the broadcast-spawning apodid *R. ruber*, although Menker [27] briefly indicated that this species lacked a larval phase and juveniles burrowed into the sediment.

The sea cucumber *C. laevis* is a ubiquitous endobenthic species occupying temperate and cold waters of the Atlantic and Pacific Oceans from intertidal to subtidal depths [45,46]. It falls on the smaller end of the apodid size spectrum, growing up to approximately 15 cm (extended length, ~6 cm contracted length). Beyond the initial examination of its anatomy by Clark [45], few other studies have included *C. laevis*, the most comprehensive being that of Ezhova et al. [23], which focused primarily on the axial complex, outlining key morphological features like the presence of a double tufted gonad rooted in the dorsal mesentery.

Interestingly, *C. laevis* is at the centre of debates on evolutionary matters. Based on morphology [47] and genetics [48], apodids have traditionally been considered a basal sister group to the other holothuroid orders; however, in their analysis of the axial complex of *C. laevis*, Ezhova et al. [23] noted that many of the findings instead position Apodida as an evolutionary intermediate between Holothuroidea and other echinoderm classes. For example, they found that *C. laevis* had five well defined water vascular canals (i.e., radial ambulacral canals), which were previously believed to be absent in apodids [49]. The same investigators demonstrated that the pores of the madreporite in *C. laevis* uniquely open into both the perivisceral coelom and the exterior environment. In most sea cucumbers, madreporic openings only lead into the perivisceral coelom [50] while in other echinoderm classes the madrepore opens only to the environment [51,52].

More recently, *C. laevis* was found to perform autotomy (self-amputation) of the posterior part of its body in response to stressful stimuli [53]. These recent insights into the unique behavioural [53], reproductive [32], and morphological [23] characteristics of *C. laevis* highlight the ecological and evolutionary importance of this species, and of apodids in general, calling for deeper studies into their fundamental life history strategies. The aim of the present study was to characterize features of the reproductive cycle and the development of *C. laevis*, including gametogenesis, spawning, embryo and juvenile development, and subsequent growth. The findings will help determine how this species, and by extension the Apodida order, aligns with the other holothuroids and echinoderms overall. *Chiridota laevis* has the potential to act as an instrumental model organism in the fields of regenerative, developmental, and evolutionary biology.

## 2. Materials and Methods

### 2.1. Collection and Holding

Individuals of *Chiridota laevis* were collected by divers in the subtidal zone (5–7 m depth) of Tappers Cove, Newfoundland and Labrador, eastern Canada (47.385961° N, 52.425309° W, *n* = 25) between June and September in 2020, 2022, and 2023. Due to collections of *C. laevis* being prohibitively difficult in Newfoundland, individuals were also collected by waders from the intertidal zone (0–2 m depth) of Middle Ground, Maine, USA (44.921542° N, 67.145950° W, *n* = 23) in April 2023. To minimize stress during transport, all individuals were hand collected and transported at low densities inside large coolers filled with seawater. Individuals collected in Maine were shipped overnight to Memorial University (St. John’s, NL, Canada) in bags of water packed in a cooler. Once in the laboratory, all individuals were held in groups of 3–5 in clear 20 L mesocosm holding tanks (267 × 394 × 216 mm) with ~3 cm of mud (collected from the subtidal zone) covering the bottom to mirror the natural habitat of *C. leavis*. To assess the suitability of various holding environments, three tanks also contained stones placed on the sediment. However, holding tanks with plain sediment were found preferable for ease of observation and were used for most of the experiments. The flow rate in the tanks was set to ~42 L h^−1^. The water temperature fluctuated naturally over the annual cycle between 0 and 9 °C, at a salinity around 35 psu, under a natural photoperiod with a peak light intensity of ≤200 lux provided through large windows. All individuals were fed on naturally deposited particulate organic matter provided by the ambient unfiltered seawater. To ensure individuals (particularly those collected in Maine) had time to acclimate to laboratory conditions, all sea cucumbers were kept in the holding tanks for a minimum period of two weeks prior to any experimental use. All individuals resumed normal feeding and burrowing behaviours during the acclimation period. Additionally, due to challenges collecting individuals of *C. laevis*, data were often gathered opportunistically (e.g., when multiple metrics could be assessed concomitantly) to maintain a robust sample size of the test population. This resulted in metrics demonstrating a range of sample sizes throughout the study.

### 2.2. Gonad Phenotype

To assess gonad morphology, individuals of *C. laevis* (*n* = 23) were dissected longitudinally; the diameter, length, and colour of their gonad tubules were recorded. For the fine assessment of the tubule branching pattern, gonad pairs (*n* = 3; male, female, and hermaphrodite) were detached at the base (i.e., attachment point to the gonoduct) and laid out on a dry surface to make both tufts and all ramifications visible and then photographed using an Olympus digital camera (TG-6 Tough Olympus, Center Valley, PA, USA) or a Leica stereoscope (using LASX software version 3.4.2.18368 and a Leica DFC 7000T camera, Leica Microsystems, Ottawa, ON, Canada). The number of tubules and primary and secondary ramifications were counted.

### 2.3. Gametogenesis

Subsets of gonad tubules from individuals of *C. laevis* were opportunistically examined using either histology or the analysis of the gametes (still in the tubules) directly through the transparent tubule wall in all months except December and May.

For histology, the isolated gonad was first preserved in neutral buffered formalin (NBF 4%), then washed in ethanol at three successive concentrations (70, 80, and 90%) and embedded in methacrylate resin. The methacrylate was left to polymerize for 12 h at 4 °C. Embedded tissues were then cut into 3 µm sections using a Leica RM2165 automated microtome. Seven tissue sections were laid on each slide and stained with Celestine Blue and Eosin–Phloxine, resulting in the nuclei being stained blue and cytoplasmic inclusions being stained pink. The sections were viewed and photographed under a light microscope (Nikon Eclipse 80i, Nikon, Tokyo, Japan) coupled to a digital camera (Olympus DP73, Olympus, Center Valley, PA, USA) and metrics were recorded, including sex, gamete dimension (diameter of oocyte and spermatozoon head), gamete location in relation to the germinal epithelium and the presence of follicular cells (for oocytes), and the location or absence of the germinal vesicle (for oocytes, ootids [32]). Measurements of gonad wall thickness were recorded between the mid- and posterior end of the tubules using histology. These metrics were used to characterize gametogenic development based on 5 stages of maturity for males and females: (1) post spawning, (2) recovery, (3) early growth (4) growth, (5) mature, adapted from Hamel et al. [30], and in females only, (6) ovulation, as described by Jobson et al. [32].

To assess the relationship between the maturity of oocytes relative to their position in the gonad, tubules (*n* = 9) were harvested from three different individuals in summer (June) and divided into 3 sections: (1) immediately adjacent to the gonad base, (2) half-way between the gonad base and the end, (3) near the end of the tubule. Oocytes (*n* = 30 minimum from each section) were measured for size. All statistics were performed in Excel or the open-source software JASP version 0.17.3.

### 2.4. Spawning and Realized Fecundity

The behaviour of *C. laevis* was monitored using infrared timelapse cameras (Brinno TLC200 Pro, Brinno, Taipei City, Taiwan) fixed over the tank mesocosms throughout the suspected spawning period of late February up to the end of March (determined in preliminary observations during multi-year holding). Videos were analyzed for evidence of spawning behaviour and to assess gamete buoyancy (speed at which they sank). Additional photos (taken using an Olympus digital camera, TG-6 Tough) of egg mats immediately post spawning allowed for the measurement of clutch dispersion and mat surface areas.

The realized fecundity, generally defined as the observed number of offspring produced, was calculated immediately post spawning. Subsamples were taken from different zones of four separate clutches using a disposable pipette, and unfertilized ootids, zygotes, and embryos counted using a Leica M205 stereoscope equipped with a Leica DFC 7000T camera and LASX software. The surface area of each subsample was also measured. It should be noted that due to the stickiness of the propagules, they were strongly attached to one another, making the surface area of each subsample variable (e.g., 30–100 mm^2^). The overall surface area of each mat was calculated and split into sections based on embryo density (high, medium, and low). To estimate the overall realized fecundity, the subsample counts (per mm^2^) for each density were multiplied by the corresponding surface area.

### 2.5. Role of Environmental Factors on Gametogenesis and Spawning

Temperatures, photoperiod, and light intensity in the holding tanks were recorded at 2 h intervals with a pendant logger (HOBO logger 64K, Onset Computer, Cape Cod, MA, USA) from January 2021 to January 2023. The moon phases were also noted during the potential spawning period of late February to the end of March. Additionally, chlorophyll-a (mg m^−3^) data were gathered through the Atlantic Zone Monitoring Program of the Canadian Department of Fisheries and Oceans at Station 27 (47.328° N, 52.352° W) located ~10 km away from the laboratory.

### 2.6. Development

Propagules from each spawning event were examined under the previously described Leica M205 stereoscope to confirm fertilization (i.e., with the elevation of the fertilization envelope or first cell division visible). All embryos were then moved to flow-through culture mesocosms (5 L) containing a layer of fine mud (1 cm) and exposed to the same environmental conditions of water flow, light, salinity, and temperature as the holding tanks (see above). Samples of embryos were collected from the culture every 1–2 d from fertilization to hatching (7 w) and monitored for changes in size and morphologies to establish developmental stages and kinetics. A minimum sample size of 10 embryos was collected at each time point but due to the sticky casing holding them together, sometimes up to 50 embryos were examined.

### 2.7. Early Growth

Once hatched, individuals were kept in the same flow-through culture conditions and monitored every week until the juvenile stage (first feeding confirmed with sediment visible in the intestine, [54]). Individuals were then monitored (photographed/videoed) using a Leica M205 stereoscope (with a Leica DFC 7000T camera Leica Microsystems, Ottawa, ON, Canada) and a light microscope (Nikon Eclipse 80i with Olympus SC50 camera Olympus, Center Valley, PA, USA) once a month until 1 y old and every 6 mo until 2.5 y old. The monitored changes included the contracted length, tentacle morphology, organ development, and the first appearance, location, and growth of ossicles. Additionally, a comparative assessment of ossicle morphology (size, shape) was performed via scanning electron microscopy (SEM; FEI Quanta 400, Thermo Fisher Scientific, Hillsboro, OR, USA) on a sample of juveniles (*n* = 20) and adults (*n* = 4). Adult ossicles (found in bundles) were removed from the body wall, rinsed with distilled water and placed in a 1.5 mL Eppendorf tube with 1 mL of sodium hypochlorite (i.e., bleach) for 1 h to allow tissues to dissolve before being centrifuged at (4000 rpm) for 30 s. The tubes were then placed in a sealed drying chamber with desiccant beads for a minimum of 32 h before mounting them on a pin stub for SEM. To prepare ossicles from small-sized juveniles (<2 mm), each complete individual was dissolved following the methods described above except it was left in sodium hypochlorite for 24 h. The resulting ossicles were mounted for SEM in the previously described way.

### 2.8. Growth Rate

The growth rate of juveniles (based on measurements of contracted length from mouth to anus in live individuals) until sexual maturity was explored using the von Bertalanffy growth function, fitted to size measurements taken at the monitoring increments mentioned above. The von Bertalanffy growth function has been previously used in sea cucumber growth studies [55,56,57,58] using the following formula:

L_t_ = L_∞_ (1 − e^−k (t − t_0_)^)

where L_t_ is the size (contracted length in mm) at a specific time, L_∞_ is the asymptotic size, K is the coefficient of the growth rate, and t_0_ is a proxy value for the time of fertilization or the time where the size would be zero. The von Bertalanffy growth function was not used to predict the maximum size for *C. laevis* as they have been shown to perform transversal scission [53] which makes size an increasingly unpredictable proxy for age in mature individuals.

## 3. Results

### 3.1. Gonad Phenotype

**Figure 1:**
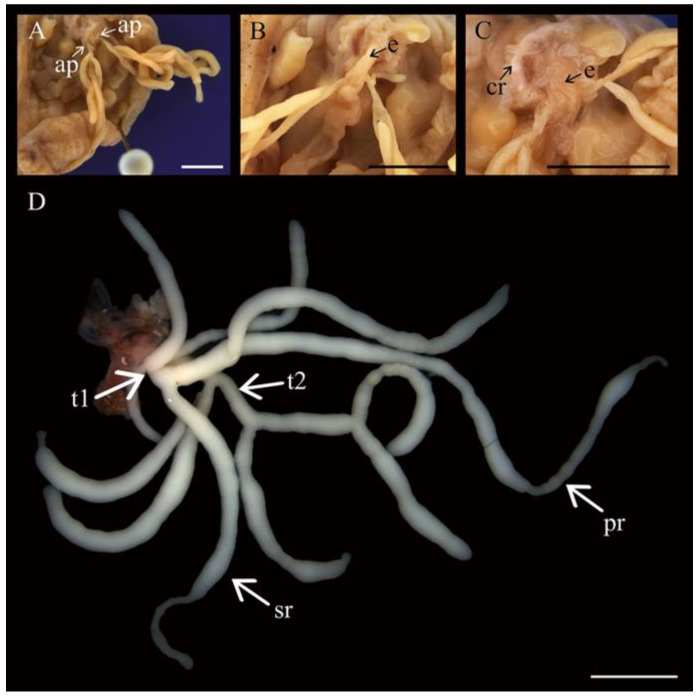
Gonad morphology of *Chiridota laevis*. (**A**) Gonad linked to the aquapharyngeal bulb by two attachment points (ap), with close-ups showing (**B**) the esophagus (e) and (**C**) the calcareous ring (cr). (**D**) Whole male gonad attached to aquapharangeal bulb showing the typical tubule arborescence and two tufts (t1 and t2) with primary (pr) and secondary ramifications (sr). Scale bars: (**A**–**C**) 1 mm, (**D**) 3.4 mm.

All individuals of *Chiridota laevis* (male, female, and hermaphrodite, see below for details) examined displayed a gonad divided into two distinct tufts, with tubules exhibiting various levels of ramification. From the main tuft branches, all tubules exhibited primary branching and most also exhibited a secondary level of ramifications (Figure 1A–D). Individuals examined in finer detail demonstrated that the male had the lowest total number of gonad tubules (*n* = 11) with nine primary ramifications and zero secondary branches. The female had 17 gonad tubules with eight primary and seven secondary ramifications. Finally, the hermaphroditic individual displayed the highest number of gonad tubules (22 total) with 17 primary branches and 3 secondary branches. The gonad tufts joined at a base to a single gonoduct rooted in the dorsal mesentery alongside the esophagus (Figure 1A–C). Gonad tubules in both sexes were largely transparent and pale in colour. Male gametes throughout development, as well as previtellogenic and early-vitellogenic female gametes, were a light beige or white colour. Large, vitellogenic, and mature female gametes developed a deepening yellow colour.

The male body length (contracted) was shorter (31.3 ± 2.9 mm) than that of females or hermaphrodites, which were similar (39.8 ± 13.7 mm and 39.2 ± 13.5 mm, respectively), although no clear statistical support emerged (*F*_2,21_ = 0.71, *p* = 0.50). Likewise, the length of gonad tubules in males (12.3 ± 7.3 mm long) and hermaphrodites (13.8 ± 10.0 mm) appeared shorter than those in females (19.9 ± 12.3 mm), without any statistically significant differences (*F*_2,18_ = 1.09, *p* = 0.36).

### 3.2. Sexuality

**Figure 2:**
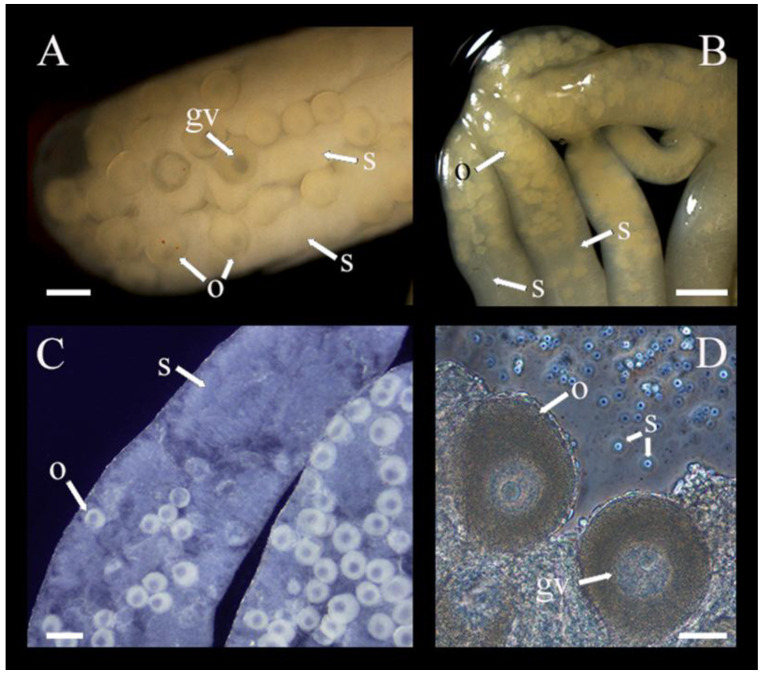
(**A**–**D**) Gonad undergoing sex change taken from individuals sampled in October and November illustrating both oocyte (o) with germinal vesicle (gv) and spermatozoa (s) in the same tubule (*n* = 17). Scale bars: (**A**) 215 μm, (**B**) 130 μm, (**C**) 190 μm, (**D**) 40 μm.

*Chiridota laevis* exhibited a mix of dieocy (with gonad tufts being either wholly female or wholly male) and hermaphroditism (with the same tubules hosting mixed male and female gametes). Globally, in the pool of individuals examined, there were ~46% females, 16% males, and 38% hermaphrodites, although there were seasonal shifts in these proportions. In winter (December–February) 33% of individuals were males, 33% were females, and 33% were hermaphrodites. In spring (March–May), males represented 17% of the population, females 50%, and hermaphrodites 33%. In summer (June–August), males represented 10%, females 70%, and hermaphrodites 20%. Finally, in the fall, (September–November) males represented 17%, females 25%, and hermaphrodites 58% (Figure 2A–D).

In hermaphroditic individuals, oocytes were mostly found at the anterior end of the tubules; their sizes ranged from 80 to 250 μm in diameter and they were beige to yellow (Figure 2C,D). Oocytes gradually diminished in density towards the mid- and posterior ends of the tubules and were sometimes entirely absent (Figure 2). Inversely, spermatozoa were less dense in the anterior section of the hermaphroditic tubules and filled the spaces between the decreasing number of oocytes in the mid- and posterior sections. No nutritive phagocytes were noted. When hermaphroditic gonad tubules were exposed to seawater under the microscope, spermatozoa were activated and could be seen swimming between oocytes, creating visible currents (Supplementary Video S1).

### 3.3. Reproductive Cycle

#### 3.3.1. Oogenesis and Ovarian Maturation

**Figure 3:**
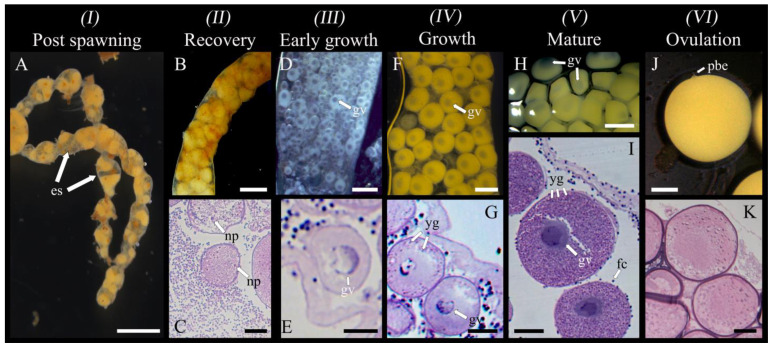
Oogenesis in *Chiridota laevis*, stages 1–6 (*n* = 17). (**A**) Gonad in post spawning (partial release) with tubule constrictions and empty spaces (es). (**B**,**C**) Gonad in recovery; ootids starting to be degraded by nutritive phagocytes (np). (**D**,**E**) Gonad in early growth, holding small and medium pre-vitellogenic oocytes (gv = germinal vesicle). (**F**,**G**) Gonad in the growth stage, holding early-vitellogenic oocytes and medium vitellogenic oocytes; early formation of yolk globules (yg, stained dark purple). (**H**,**I**) Mature gonad, holding large, vitellogenic oocytes and well-defined follicle cells (fc). (**J**) Ovulation stage, with gonad tubules holding ootids that show the second polar body expulsion (pbe). (**K**) A thick casing is visible around the ootids devoid of a germinal vesicle. Scale bars: (**A**) 1 mm (**B**) 450 μm (**C**) 72 μm (**D**) 120 μm, (**E**) 35 μm, (**F**) 180 μm, (**G**) 55 μm, (**H**) 350 μm, (**I**–**K**) 90 μm.

**Figure 4:**
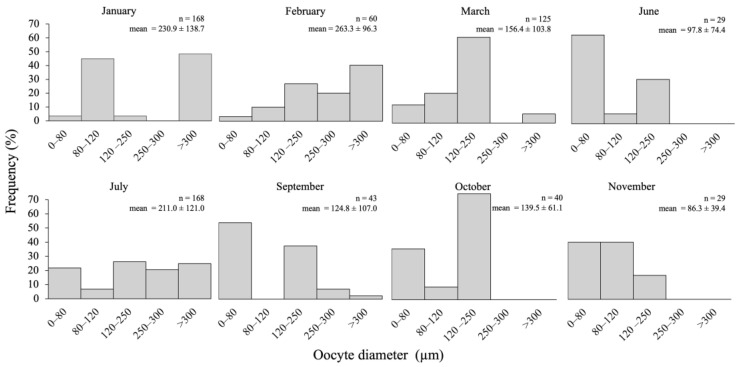
Annual variation in oocyte diameter (μm) in *Chiridota laevis* (*n* = number of oocytes; mean ± standard deviation).

Overall, based on oocyte size structure and histology, oogenesis appeared to be asynchronous inside a gonad tubule, with oocytes at all stages of development (except ootids) found year round in a clear sequence of maturity along a tubule. In tubules in the growth to mature phase of development (see below), oocytes held in the posterior (i.e., farthest from the gonad base and gonoduct) and middle sections of gonad tubules were in the most advanced stages of development and significantly smaller oocytes were held in the anterior portion (*F*_2,267_ = 6.83, *p* = 0.001). Just before and at the time of spawning, most female gametes were mature or already ovulated (inside the gonad tubules), with a minority of early-vitellogenic oocytes in the anterior section still present. Accounting for the spatial patterns and asynchrony in oogenic development (i.e., using mid- and posterior sections of the tubules containing oocytes likely to be involved in the next spawning), the gametogenic stages of tubules were classified as follows:

*Post spawning*: Occurring immediately after spawning; gonad wall showing characteristic constrictions; empty spaces in the lumen, with some residual ootids (yellow in colour) remaining (Figure 3A). This stage was mostly observed in March when the largest oocytes and ootids decreased in abundance abruptly (Figure 4).

*Recovery*: Tubule wall thin (6.4 ± 2.1 μm) and convoluted; tubules primarily characterized by visibly degrading residual oocytes (yellow in colour) with the presence of associated nutritive phagocytes (Figure 3B,C).

*Early growth*: Dominance of pre-vitellogenic oocytes (<30 μm, translucent, no globular yolk protein in the cytoplasm) closely associated with germinal epithelium; the presence of scattered early vitellogenic oocytes (30–80 μm, whitish) in the lumen; nutritive phagocytes and residual ootids no longer visible (Figure 3D,E). From July, small oocytes dominated the size structure (Figure 4).

*Growth (IV)*: Tubule wall at maximum thickness (18.8 ± 9.9 μm) and still convoluted; dominance of vitellogenic oocytes between 80 and 120 μm (beige in colour, with first signs of visible globular yolk proteins around the periphery of the oocyte) and few measuring 120–250 μm (faint yellow, with globular yolk proteins spreading throughout the cytoplasm) visible inside the lumen (Figure 3F,G). For September to November, the gonad tubules harboured small and large oocytes sometimes in two distinct cohorts (Figure 4).

*Mature (V)*: Tubule wall beginning to thin (16.4 ± 19.6 μm) and was no longer convoluted; dominance of large vitellogenic oocytes ≥ 250–350 μm (deeper yellow, with globular yolk proteins spreading throughout the entire cytoplasm; Figure 3H,I). Beginning in January and culminating in February–March, the proportion of large oocytes increased to progressively reach a maximum, probably up to the spawning period in March (Figure 4).

*Ovulation (VI):* Thin tubule wall (7.9 ± 3.6 μm) no longer convoluted; dominant presence of largest vitellogenic oocytes (~350 μm), most having completed ovulation (the disappearance of follicle cells); GVBD and polar bodies expulsions (i.e., ootids having completed final meiotic maturation), surrounded by a now-visible casing (Figure 3J,K).

#### 3.3.2. Spermatogenesis and Testis Maturation

**Figure 5:**
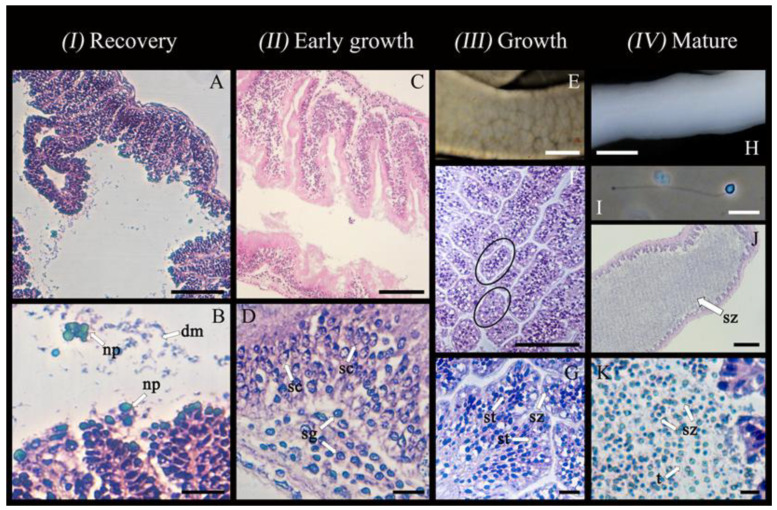
Spermatogenesis in *Chiridota laevis*, stages 1–4 (*n* = 6). (**A**,**B**) Gonad in post-spawning recovery, holding spermatozoa being degraded by nutritive phagocytes (np) and already degraded material (dm). (**C**,**D**) Gonad in early growth, holding spermatogonia (sg) and spermatocytes (sc). (**E**–**G**) Gonad in the growth stage, showing external view of gonad and internally holding spermatids (st) and spermatozoa (sz) partitioned in sections (highlighted by circles). (**H**) Mature gonad, showing external view of gonad; (**I**) a spermatozoon with visible tail (t); (**J**,**K**) tubules packed with spermatozoa (sz). Scale bars; (**A**,**B**) 85 μm, 20 μm, respectively, (**C**,**D**) 140 μm, 20 μm, respectively, (**E**) 12.5 mm, (**F**,**G**) 150 μm, 20 μm, respectively, (**H**) 11 mm, (**I**) 18 μm, (**J**) 344 μm, (**K**) 44 μm.

Contrary to oogenesis, the various stages of spermatogenesis were uniform along the entire gonad tubule. Four testis maturation stages were developed and used:

*Post spawning and recovery (I)*: Empty tubules with residual gametes not clearly observed/confirmed (only partial spawning); tubule wall at its thinnest (15.8 ± 6.3 μm) with slight convolutions visible; residual spermatozoa being degraded by nutritive phagocytes (Figure 5A,B).

*Early growth (II)*: tubule wall thickening (37.0 ± 27.2 μm) with deep convolutions; layers of spermatogonia (4–6 μm cell diameter; innermost layer) and developing spermatocytes (6–8 μm cell diameter; second layer) packed along the germinal epithelium; lumen largely empty; nutritive phagocytes and residual spermatozoa absent (Figure 5C,D).

*Growth (III)*: further thickening of tubule wall (39.6 ± 3.7 μm) with less convolutions; lumen starting to fill with spermatids (elongated head sizes ranging from 6 to 8 μm) partitioned in separate, membrane-bound compartments ranging widely in size from 55 × 27 μm to 481 × 40 μm (Figure 5F,G); stage dominated by spermatids but some early spermatozoa also visible (Figure 5G).

*Mature (IV)*: tubule wall remaining at its thickest (39.5 ± 5.9 μm) with small convolutions reappearing; lumen filled with spermatozoa (head sizes ranging from 10 to 12 μm; visible nucleus and flagellum; Figure 5H–K) without partitions present as seen in the growth stage.

### 3.4. Spawning

**Figure 6:**
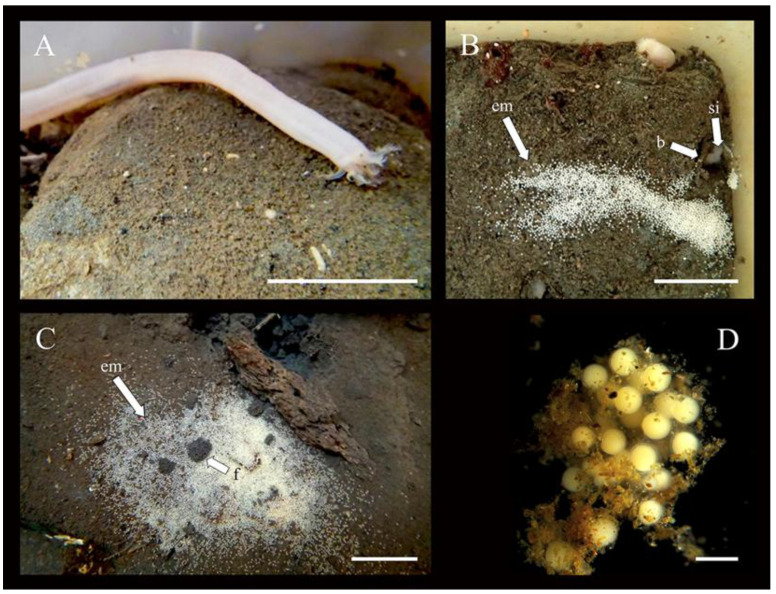
(**A**) Individual of *C. laevis* climbing up onto a stone before broadcasting ootids. (**B**,**C**) Spawned ootids forming mats next to burrows and physical supports like stones in mesocosm. Labels: em = egg mat, b = burrow, si = spawning individual, f = fecal matter. (**D**) Group of ootids/embryos surrounded by a casing adhering to each other and partly covered by fine sediment. Scale bars: (**A**–**C**) 12 mm, (**D**) 450 μm.

Before releasing gametes, individuals emerged from the sediment, raising the anterior half to two-thirds of their body out of the burrows. At the same time, they were typically seen pressed against hard substrata (e.g., stones or mesocosm walls) as they extended all tentacles and waved them through the water (Figure 6A), holding this posture for up to 4 h. Ootids were released in multiple sessions lasting from 45 to 200 s. Immediately after contact with seawater, the outer casing of the ootids became sticky allowing them to attach to one another and a few (<1%) even became temporarily stuck to the mesocosm walls. They were negatively buoyant and fell rapidly (<15 s) to the sediment, adhering both to the mud and to each other in the form of a mat with variable ootid density (i.e., the mat was most dense close to the spawning individual becoming gradually less dense with farther dispersal; Figure 6B,C). The mats varied widely in both shape (e.g., circular to elliptical; Figure 6B,C) and surface area (38 to 389 cm^2^). Only a small portion of the ootids (<1%) strayed away from the mat as far as ~40 cm. Embryos within the mat remained attached to each other for the first month following spawning (Figure 6D; see also development details below). The realized fecundity was estimated to be 15 263 ± 6 016 offspring per clutch.

### 3.5. Correlation of Reproductive Cycle with Environmental Factors

**Figure 7:**
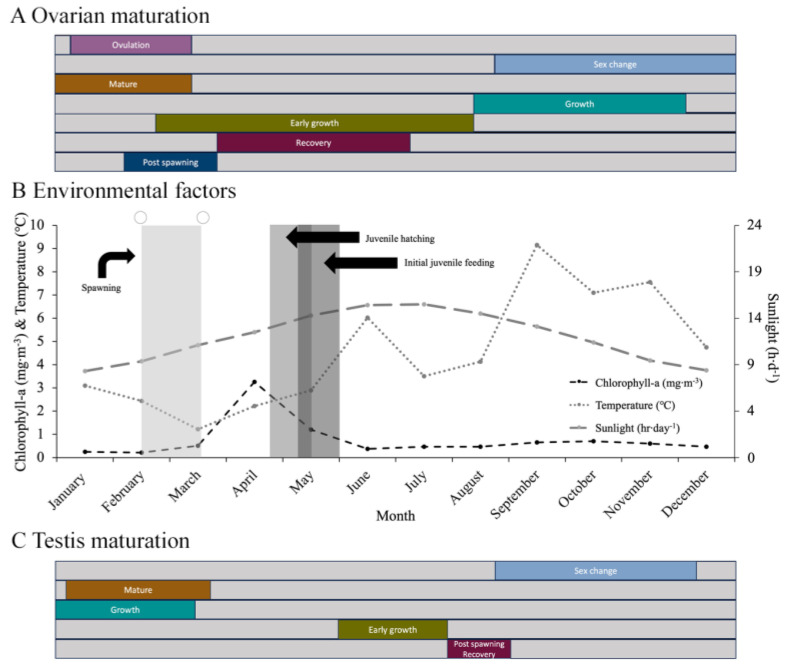
(**A**) Annual cycle of ovarian maturation. (**B**) Spawning, hatching, and initial feeding of juvenile *Chiridota laevis* related to temperature (°C), concentration of chlorophyll-a (mg m^−3^), photoperiod, and lunar cycle (○ = full moon). (**C**) Annual cycle of testis maturation.

Individuals with gonads in the post-spawning stage (directly documented in females) were seen in February or March when temperatures rose from the lowest temperatures in the annual cycle, from 1 to 2.5 °C, and daylight hours increased from 9.5 to 11.5 h d^−1^ (Figure 7A,B). Chlorophyll-a levels remained low (around the baseline winter level of 0.37 mg m^−3^) during this time. Moreover, during the year with the lowest temperatures (1.1 °C) three spawning events were recorded. This is contrary to the other two years where only one annual spawning occurred, and seawater temperatures remained above 2.2 °C. No relationship between spawning and the lunar cycle was detected.

Females with a gonad in early growth were found primarily between spring and early summer (March to June) when temperatures ranged from 1 to 6 °C and daylight from 11 to 16 h d^−1^ (Figure 7A,B) and when the chlorophyll-a levels peaked (Figure 7). However, males with testes at the same stage were seen later, from June to July, as temperatures ranged from 3.5 to 6 °C and daylight from 15.5 to 16 h d^−1^. The growth- and mature-stage oocytes in late vitellogenesis and increased number of spermatozoa coincided with a period beginning in January and ending close to the time of spawning (late February to early March). Intragonadal ovulation (the completion of meiosis to produce ootids) was seen during the same period (Figure 7). Hermaphroditism (i.e., male and female gametes mixed in the same gonad tubule) was primarily documented from September through to November, coinciding with a major temperature increase from 5 °C toward the peak annual value around 9 °C. The fall months also saw the recruitment of oocytes in the early and growth phases.

### 3.6. Development

**Figure 8:**
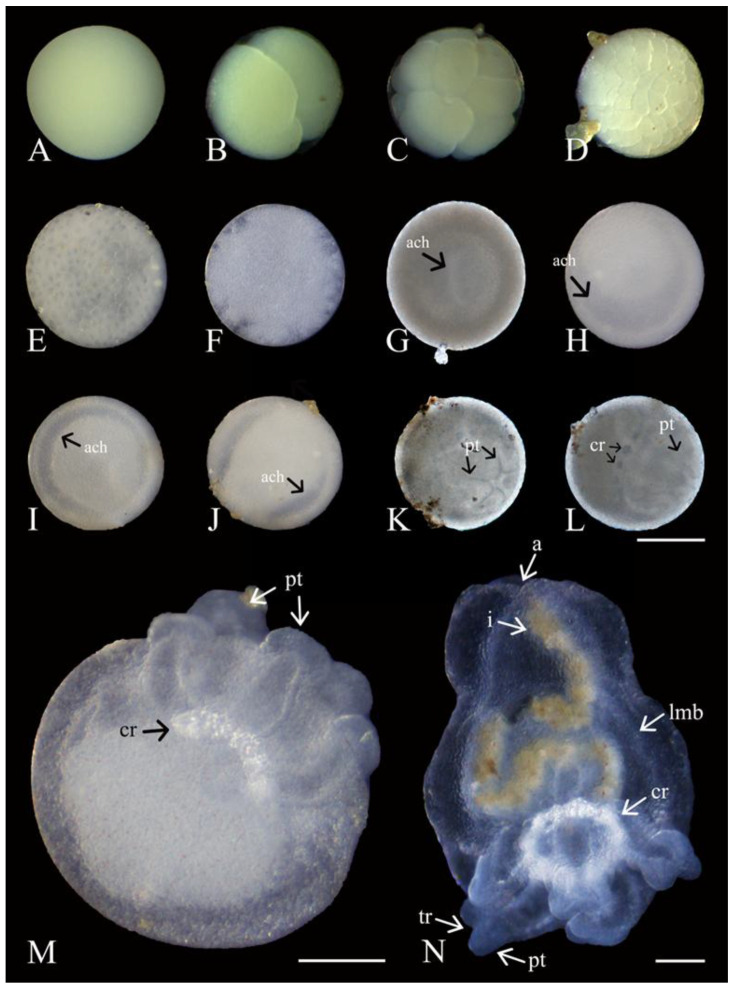
Development of *Chiridota laevis*. (**A**) Unfertilized; (**B**–**E**) early cell division (days 1–6); (**F**) blastula; (**G**–**J**) gastrulation (ach = archenteron; days 8–40); (**K**) first evidence of primary tentacles (pt; day 43) (**L**); development of tentacles (pt) and calcareous ring (cr; day 45); (**M**) post hatching from casing into pentactula with five tentacles (pt) and calcareous ring (cr) clearly defined (day 49); (**N**) juvenile with longitudinal muscle bands (lmb), tentacles (pt), and developing ramifications (tr), anus (a), intestine (with brownish content; i), and calcareous ring (cr) visible (day 75). Scale bars: (**A**–**L**) 150 μm, (**M**) 75 μm, (**N**) 100 μm.

The ootids (ready to be released) were ~350 µm in diameter (see oogenesis above), yellow in colour (partly translucent), and rich in vitellus at the time of broadcast spawning. They demonstrated a smooth surface surrounded by a thin transparent casing that became sticky post release (Table 1; Figure 8A).

Following spawning (<3 h), ootids and zygotes were indistinguishable. However, within the first 12 h, stages from two to eight cells (equal-size holoblastic blastomeres) were observed (Figure 8B,C). After 24 h, embryos from 8 to 32 cells were visible. On day 3, embryos demonstrated between 32 and 128 cells, and blastomeres continued to be of similar sizes (Figure 8D). By days 4–7, the surfaces of the embryos at the blastula stage started appearing more uniform, with blastomeres becoming barely identifiable (Figure 8E,F). At this point, the remaining unfertilized ootids (~20% of released gametes) mixed with the developing embryos began to degrade. Among the developing embryos, the blastopore became visible after 7 d, and gastrulation progressed from days 8 to 40, with the appearance of the archenteron being the primary visible change during this timeframe (Table 1; Figure 8G–J). The first indication of primary tentacle development came on day 43 (Figure 8K) and the calcareous ring became visible on day 45 (Figure 8L). The embryos at this stage had not increased in size and were still measuring ~350 μm. No movement or ciliation of the embryos were recorded.

Hatching occurred on day 49, with pentactulae using their five tentacles to break open the casing (Figure 8M). Hatching occurred in mid-April following a spike in chrolophyll-a concentration (3.25 mg m^−3^). Although they immediately began burrowing through the sediment using their tentacles, there was still no functional digestive tract (Figure 8M). These pre-juveniles displayed an almost round body with the length of the tentacles making up about one third of the overall length of the organism (Figure 8M). There was no obvious differentiation of internal organs in the aboral region; however, the calcareous ring was more defined. After about 60 d post spawning and 7 d after hatching, the tentacles were capable of adhering to hard substrata. From days 60 to 70, the body of pre-juveniles elongated into an oval shape with defined longitudinal muscle bands (Table 1; Figure 8N) increasing the capacity for body contraction and expansion. By day 68, a looped intestine became distinctly visible, running from mouth to anus. Around the same time, the five tentacles began to show the first signs of ramifications with a split appearing to create two digits. On day 75, sediment became visible throughout the digestive tract, marking the onset of the juvenile stage (Figure 8N). The release of fecal matter was also first observed. The juvenile stage (in May) occurred as chrolophyll-a concentration started dropping to baseline levels (0.37 mg m^−3^).

### 3.7. Juvenile Growth

**Figure 9:**
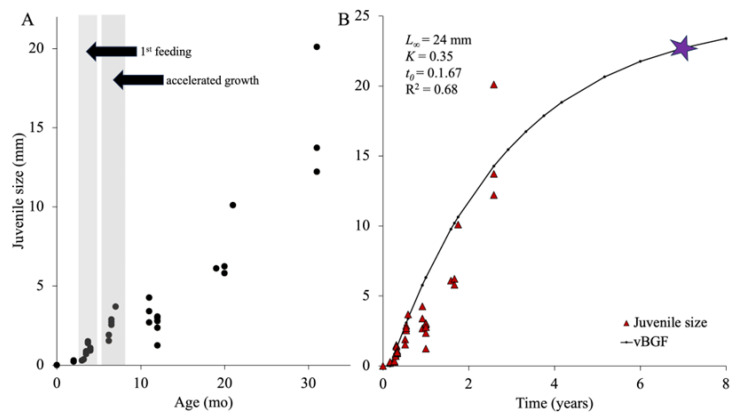
(**A**) Juvenile growth across the first 31 mo of development with the first feeding and period of accelerated growth highlighted in grey. The growth spurt occurred during the warmer months of August to November. (**B**) von Bertalanffy growth function fitted to growth data of juveniles of *Chiridota leavis* over the first 2.5 y of growth. The star indicates estimated age at reproductive maturity (~7 y). L_∞_ = asymptotic size of a species, K = coefficient of the growth rate, and t_0_ = a proxy value for the time where size would be zero (*n* = 40, 1 individual per point).

**Figure 10:**
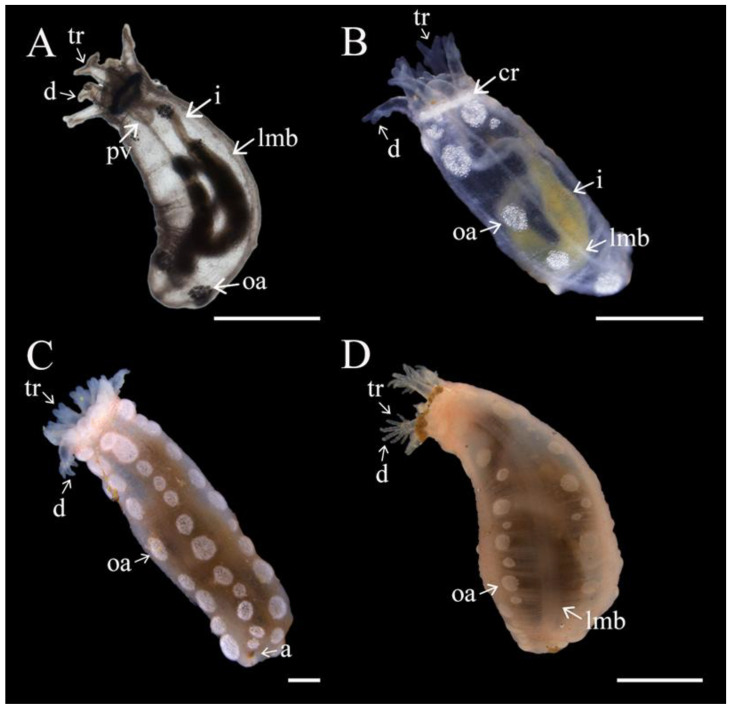
Development and growth of *Chiridota laevis*. (**A**) After 7 mo with visible Polian vesicle (pv), tentacle ramification (tr), two digits (d), ossicle aggregation (oa), intestine (i), and longitudinal muscle bands (lmb); (**B**) 1 y with additional tentacle ramification (tr) creating 4 digits (d), ossicle aggregations, intestine (i), calcareous ring (cr), and longitudinal muscle bands (lmb); (**C**) 1.5 y showing tentacle ramifications (tr) creating 4 digits (d) and ossicle aggregations; (**D**) 2.5 y showing additional tentacle ramification (tr) creating 8 digits (d), ossicle aggregations, and longitudinal muscle bands (lmb). Scale bars: (**A**–**C**) 1 mm, (**D**) 3.6 mm.

The body length (mouth to anus) of pre-juveniles remained consistent with ootid/egg size at ~350 μm for about 3 mo (see above; Table 1; Figure 9A). From then, as they began to feed, juveniles tripled in size to reach between ~740 and 950 μm in length by the end of their first week, ~900–1074 μm by 4 m, ~1.52–2.87 mm by 6 mo, and ~3.60 mm by 7 mo (Figure 10A).

The increasing size variability among individuals of the same cohort continued during “growth spurts” which occurred during warmer months (August–November; Figure 9A) with some individuals doubling in size within a month. The growth curve tapered off subsequently, with individuals remaining the same size throughout the colder winter and spring months (e.g., ~2.35–3.60 mm at the end of year one; Figure 10B). After 1.5 y, as the temperatures warmed again (September–October), the size of juveniles double to ~5.8–6.1 mm. As temperatures cooled in the winter, growth rates slowed again with juveniles reaching ~10 mm by year 2. There was a less noticeable “growth spurt” in the second year with juveniles growing up to ~14 mm by 2.5 y of age (Figure 10C,D). The von Bertalanffy growth function predicted that, assuming that individuals reach sexual maturity around 24 mm contracted body length (first appearance of a gonad with mature gametes), it would take this species between 6 and 7 y to reach it (Figure 9B).

### 3.8. Ossicle and Tentacle Development

**Figure 11:**
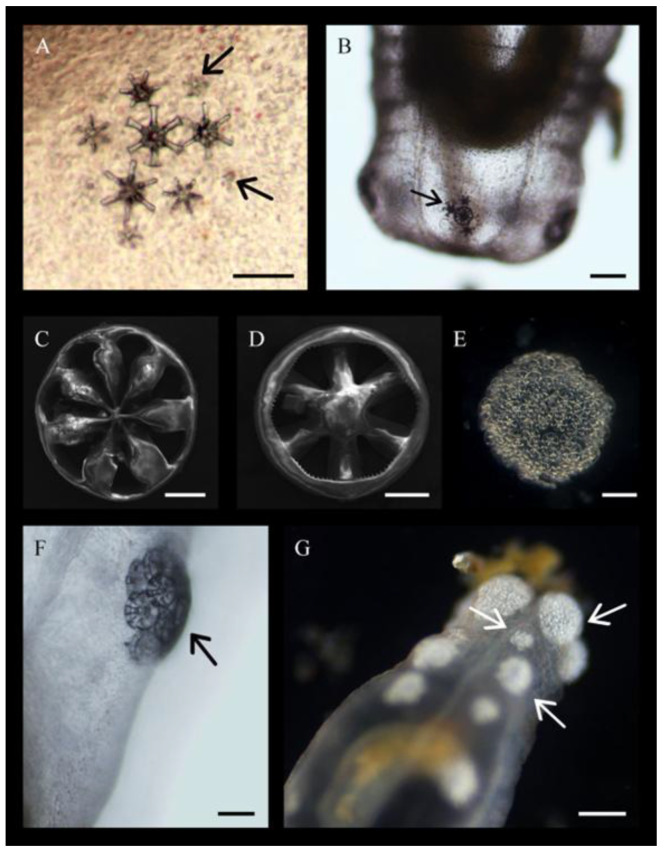
Ossicle development in *Chiridota laevis.* (**A**) Arrows indicate initial ossicle formation at the posterior end. (**B**) Aggregation of ossicles including one complete and three incomplete wheel ossicles at the posterior end. Arrow indicates beginning of full circular connection. (**C**) One side of a fully formed ossicle. (**D**) Opposite side of a fully formed ossicle. (**E**) Mature aggregation of ossicle from an adult. (**F**) Early formation of ossicle aggregation in a juvenile (arrow). (**G**) Dispersion of ossicle aggregates in rows across the body wall (indicated by arrows). Scale bars: (**A**) 56 µm, (**B**) 88 μm, (**C**) 31 μm, (**D**) 28 μm, (**E**) 115 μm, (**F**) 62 μm, (**G**) 226 μm.

At 6 mo post spawning, the first signs of ossicle development were noted in five locations directly between longitudinal muscle bands at the posterior end of the body. They appeared as a small dark spot, almost indistinguishable from the surrounding tissue (Table 1; Figure 11A). From this, 6–7 spines emerged and, after growing to about 50 μm in length, additional calcareous deposits created a “cap” running horizontally to the spines (Figure 11B; arrow). This cap eventually connected to create a shape reminiscent of a wheel with six or seven spokes (Figure 11C,D) aptly known as a wheel ossicle. The first complete wheels formed immediately adjacent to one another, increasing in numbers, building around and on each other to create an aggregate (Figure 11E,F). Within two weeks of the first ossicle aggregates, wheels began to form at the anterior end of the body (Figure 11G). At 12 mo old, more ossicle aggregates (4–7 aggregates up to 330 μm across) developed in a single straight row directly between two sets of longitudinal muscles. Notably, ossicle development was concentrated dorsally on the body with almost no ossicles appearing on the other side. At 1.5 y, the number of ossicle aggregates (up to 630 μm across) ranged from 5 to 11 per row and, by 2 to 2.5 y, they numbered 16–21 per row and measured up to 836 μm across.

**Figure 12:**
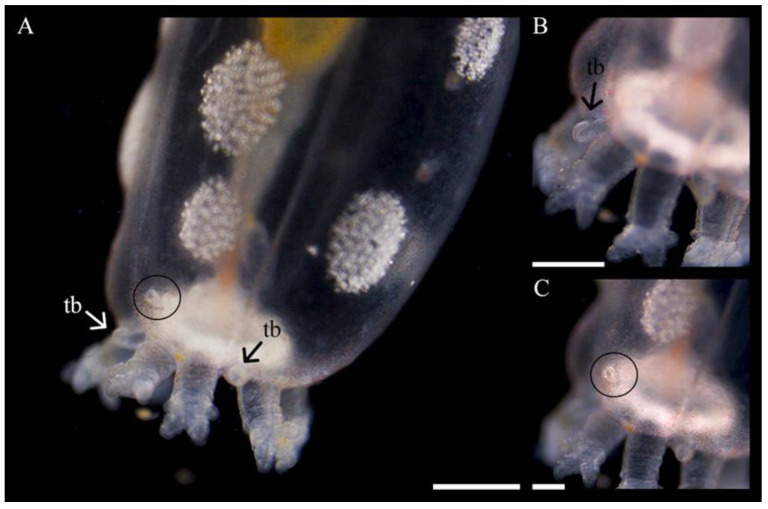
Development of new tentacle buds (tb) and what might be the gonopore/genital papilla or pore canal (circled) in a 1.5 y old individual of *Chiridota laevis*. Scale bars: (**A**) 490 μm, (**B**) 370 μm, (**C**) 245 μm.

Over the same period, the tentacles also developed in both number and complexity (ramification). At 6 mo old, the second ramifications appeared on the five primary tentacles (the first ramification had occurred at the pre-juvenile stage; see above), thus creating tentacles with four digits at the base of the first split. Concurrently, one additional tentacle bud (rounded, unramified; Figure 12) was added, bringing the total to six tentacles; when juveniles reached 12 mo, the number increased to 7–8 tentacles, each showing four digits (Table 1). Juveniles still had a transparent body wall at this stage. At 14 mo, the body wall showed the first sign of pink and red pigmentation, while the number of tentacles ranged from 9 to 10, all with four digits at 1.5 y, and a third set of ramifications appeared at the base of the previous split, creating six digits on all tentacles. At 2.5 y, there were 10–12 tentacles, each with eight digits.

What was assumed to be the gonopore/genital papilla (but could also be a pore canal, [23]) was first noted dorsally in juveniles 1.5 y old, between and slightly below two tentacles, and generally on the same side of the body as the rows of ossicle aggregations (Figure 12).

## 4. Discussion

*Chiridota laevis* stands out among cold water holothuroids for its unusual combination of reproductive adaptations, including sequential hermaphroditism, intragonadal ootids, negatively buoyant and sticky embryos, direct development, and slow growth rate.

### 4.1. Sexuality

While there are several types of hermaphroditic apodids (e.g., self-fertilizing, reciprocal, sequential), the data presented here supports that *C. laevis* is of the sequential type. Hermaphroditic individuals were primarily found in the fall, whereas the population was predominately gonochoric the rest of the year, as opposed to the year-round presence of ovotestes characterizing permanent hermaphrodites (e.g., self-fertilizing species like *S. hydriformis*, [36]). However, it was not clear from the gametogenic data whether the sex change sequence in *C. laevis* was from male to female, i.e., protandry as in *Leptosynapta clarki* [17,21], from female to male (protogyny), or whether it was bidirectional.

In an effort to clarify this question, we can compare the average body sizes of males, females, and hermaphrodites to test the size advantage hypothesis proposed by Ghiselin [59]. This model suggests that, as oocytes require more space per gamete than spermatozoa, incremental body size is an indicative factor of sex change from male to female (i.e., protandric hermaphroditism). In *C. laevis* the body size of hermaphroditic individuals was comparable to that of females and almost 25% larger than that of males, providing evidence of protandry. A similar protandric strategy has been described in the apodid *L. clarki* where all juveniles begin as male but some males in the population transition to females upon reaching a critical size (200–400 mg overall weight, [21]). This strategy was proposed to allow *L. clarki* to maintain a 1:1 sex ratio in populations [21]. The present study on *C. laevis* showed that the male to female ratio immediately following sex change (i.e., during the spawning season) was 1:1, irrespective of the year studied, suggesting that sex change could also be important for maintaining the appropriate sex ratio among individuals of its populations. However, the conclusion that protandry occurs based on body size alone should be treated with caution as spontaneous transversal scission (autotomy) is known to occur in *C. laevis* [53]). The fact that autotomic individuals can become smaller means that female to male or even bidirectional sex change cannot be ruled out. Autotomy may explain why even though females and hermaphrodites both averaged a contracted length of ~39 mm, they exhibited a wide range of sizes (23–55 mm). More research is needed to confirm the sex change sequence in *C. laevis* and to clarify whether autotomy plays any role in it.

When present, the hermaphroditic gonad of *C. laevis* contained a mix of male and female gametes where the female gametes (all immature and ranging from pre-vitellogenic to medium-sized vitellogenic oocytes) were housed primarily at the anterior end of the tubule. The female gametes gradually disappeared towards the middle of the tubule and were replaced by mature male gametes (spermatozoa) densely packed in the posterior region of the hermaphroditic tubules. As the synthesis of new gametes seems to occur in the anterior section of the gonad (especially clear in females), the appearance of spermatozoa in the posterior suggests that its development was initiated first (filling the posterior). The appearance of oocytes in the anterior suggests that oogenesis began at a later stage. The partitioning of male and female gametes in this way supports the possibility that *C. laevis* is a protandric hermaphrodite that switches from male to female.

It is also possible that the sex change in *C. laevis* is initiated seasonally as the presence of simultaneous hermaphrodites was most common during the fall months which also corresponded to the highest water temperatures of the year. Warmer temperatures have been shown to support more efficient metabolic processes, subsequently facilitating growth [60], and it may help *C. laevis* undergo the costly gametogenic shift in initiating oogenesis [61]. In contrast to the ~3 mo sex change process seen in *C. laevis*, sex change in *P. refescens* and *P. ooplax* is reportedly more rapid (completed over ~2 w) and related to the lunar cycle [15,20]. Interestingly, based on preliminary results, *C. laevis* is able to perform transversal scission and subsequently regenerate in warmer months, but not in the winter further linking temperature and increased nutrient availability in the environment to sustain more important metabolic processes. Moreover, Dobson et al. [62] suggested that the same mechanism of nutrient translocation used in tissue regeneration is also used in gametogenesis, offering some insight into why these costly behaviours coincide seasonally.

### 4.2. Gametogenesis

Similarly to the hermaphroditic apodids *L. clarki* and *S. hydriformis* [17,29], the female gonad of *C. laevis* holds multiple cohorts of oocytes at one time. Additionally, the ovotestis of *S. hydriformis* have demonstrated gametogenic partitioning comparable to *C. laevis* where gametes of the same developmental stage appear segregated in the same position across tubules. This asynchronous development allows *S. hydriformis* to release a cohort of mature oocytes while retaining the immature oocytes for future spawning seasons. Inversely, *C. laevis* seems to initiate oocyte development in the anterior portion of the gonad tubule (this section consistently held the youngest and smallest oocytes) with gametes moving towards the posterior end as vitellogenesis advanced. However, at the time of spawning most oocytes within the gonad had reached maturity. As opposed to releasing cohorts of oocytes like *S. hydriformis*, it seems likely that *C. laevis* releases the minority of anteriorly held, still-immature oocytes concomitantly during the spawning of posteriorly held ootids. Following this, all remaining gametes (oocytes and ootids) are apparently resorbed by coelomocytes in the gonad during the recovery phase [30,63].

### 4.3. Spawning

The final maturation (i.e., ovulation, [32]) and broadcast of gametes in *C. laevis* occurred in the late winter (February–March) of all three years. The repeated occurrence of winter spawning events indicates that, as is common within the phylum Echinodermata [13], external factors likely play a role in triggering and maintaining the consistency of this behaviour [64]. The year with the highest number of spawning events and highest fertilization rate was also the year with the coldest water temperatures, while other metrics like photoperiod, chlorophyll-a, lunar phases, and holding conditions remained the same across years or showed no clear correlations. This suggests that temperature may be the primary factor influencing the spawning of *C. laevis*, as also reported in the apodid *P. rufescens* [15] and the sympatric sea star *Henricia lisa* [65]. The low sea water temperature may act as a proximate spawning cue designed to optimize development since embryos did not hatch until after the phytoplankton bloom, allowing time for the particulate organic matter (the most probable food source of *C. laevis*) to settle to the benthos. A similar pattern was seen in three other species of apodid sea cucumbers (*Opheodesoma grisea*, *Synapta maculata,* and *Patinapta taiwaniesis*) off the coast of southern Taiwan where spawning occurred mid-summer, immediately prior to increases in water temperature and subsequent peaks in chlorophyll-a levels [14]. Chao et al. [14] also suggested that food availability may be a key determinant for the spawning season.

While some other holothuroid species exhibit negatively buoyant oocytes (e.g., *Cucumaria lubrica* and *C. pseudocurata*, [66]) the demonstration of a sticky casing adhering eggs or embryos together post spawning is more rare. The tropical sea cucumber *Holothuria floridana* spawns negatively buoyant, sticky oocytes similar to those seen in *C. laevis*, but they are found scattered on various substrata including seagrasses and the adult themselves for a maximum of 6 d before reaching the juvenile stage [67]. In *C. laevis*, the negatively buoyant eggs and their stickiness could serve as an adaptation to its muddy/sandy habitat and long development time before hatching (~49 d). The physical or chemical nature of the egg casing might repel predators whereas the mat formation may prevent the developing offspring from sinking into the substratum or drifting with the current, as this species is commonly found in the intertidal zone.

Fecundity is not well documented in broadcast-spawning apodids, offering little basis for comparisons to the realized fecundity seen here in *C. laevis* (~15,000 ootids released by each spawner). It is higher than that of the intraovarian apodid brooder *L. clarki* which produces an average of 174 offspring [28]. It is likely that unprotected embryos are more vulnerable than internally brooded ones, calling for higher fecundity to maintain population levels [68].

### 4.4. Development

The development of *C. laevis* from ootid to pentactula (~49 d) was slower than typical for temperate cold apodids and other orders of cold water sea cucumbers. For example, the embryos of the internally brooding, direct-developing apodid *L. clarki* reportedly take from 10 to 14 d to reach the pentactula stage [28]. It is also notable that *C. laevis* does not demonstrate a complex larval phase, instead displaying direct development by hatching into a pentactula. Although limited, the previous literature on apodid development has documented that both brooding and free-spawning apodids either demonstrate an indirect larval phase (e.g., *C. gigas*, *C. rotifera*, *L.inhearens*, *Protankyra brychia*, *S. maculata*, *Opheodesoma grisea*, *P. taiwaniesis*, and *P. fusca*, [14,35,37,38,69]) or at least some larval attributes. For instance, the internally brooding apodid *S. hydriformis* may not exhibit the aucricularia and doliolaria stages typically seen in holothuroids but still hatch as a “modified” auricularia larva [29]. In contrast, *C. laevis* did not show any modified auricularia or doliolaria and hatched as a pentactula. The size of the ootids, ~350 µm in diameter, and the absence of a feeding larva support lecithotrophic development in *C. laevis* differing from planktotrophic apodids like *C. gigas* and *P. rufescens* whose mature oocytes measure < 115 μm [14,38]. As *L. clarki*, the other apodid species demonstrating direct development and a large egg size (~200 μm), is considered matrotrophic, to our knowledge this is the first documented case of external direct development in apodids.

From the time the propagules of *C. laevis* emerged from the sticky casing until the confirmation of first feeding (anus opening and food items visible in the digestive tract) their size remained the same. The development of the internal organs was likely fueled by energy stored in the yolk of oocytes, as is a hallmark of non-feeding lecithotrophic species [70]. Growth only began after feeding was observed, with most individuals reaching half the average adult size after 2.5 y. The slow growth rate of this species is highlighted by a comparison with the temperate apodid *L. clarki* where juveniles grow to ~21 mm and are sexually mature after one year [18]. Developing juveniles of *L. clarki* were exposed to more consistent conditions (8–11 °C, [18]) compared with *C. laevis* (~1–9 °C), which could contribute to the variable growth rate. Based on the von Bertalanffy growth function, it is estimated that *C. laevis* could not reach sexual maturity until 6–7 y of age, which is slower than *L. clarki,* as described above [18]. However, the occurrence of transversal scission in *C. laevis* [53] limits the uses of size as a precise proxy for age, so such estimates should be used cautiously.

The ossicle and tentacle development in *C. laevis* is similar to what has been noted in other apodids possessing wheel ossicles and peltato-digitate (sometimes referred to as digitate) tentacles [35,45]. As seen here, initial ossicle development occurs posteriorly in other apodids (*C. gigas*, *C. rotifera*, and *Synapta digitata*, [35,38,71]) regardless of whether the species demonstrated indirect (e.g., *C. gigas*, [38]) or abbreviated (e.g., *C. rotifera*, [35]) development. However, the pattern of ossicle formation determined in *C. laevis* (i.e., posterior ossicle development followed by anterior formation and finally a row of ossicles forming between the two points) is rarely discussed (e.g., in the genus *Chridotae*, [45]). As apodids do not have tube feet or other obvious external morphological features dictating the dorsal versus ventral orientation of an individual, orientation terms (e.g., dorsal/ventral or bivium/trivium) are not commonly used in apodids or are used to denote the location of the genital papillae and gonad (both assumed to develop dorsally, [26,35]). The results presented here show that the genital papilla (gonopore) develops in an interambulacral space also containing a full row of ossicle aggregates, although it may also be the pore canal described by Ezhova et al. [23] or a mix of both. Because of this association, we suggest that the side of the body presenting ossicle aggregates is the dorsal side and the side of the body where ossicles aggregates are absent is the ventral side. As it is difficult to see the genital opening in apodids without a microscope, using ossicle aggregates could provide a reliable means of defining dorsal and ventral orientations. Moreover, the purpose of wheel ossicle aggregates is not well established; however, some researchers have posited that as these ossicles create bumps on the surface of the epithelium (akin to those seen in *C. laevis*) they can be used as traction to help move individuals burrow through the sediment (e.g., *C. rotifera*, [11,72]). It is likely that the ossicle aggregates in *C. laevis* serve a similar role.

Juveniles of *C. laevis* began with five primary tentacles at the pentactula stage and underwent two tentacle ramifications before any additional tentacles developed. This is comparable to what has been documented in juveniles of *C. rotifera* and *S. hydriformis* [35,45]. By the end of our observation period (2.5 y) juveniles had developed between 10 and 12 tentacles, which is slightly less than the maximal amount noted in adults of this species (up to 15; personal observation). Judging by the speed of previous tentacle development (about 6 mo between tentacle formations) we can estimate that juveniles of this cohort will need 1.5–2.5 more years before all tentacles will be formed.

## 5. Conclusions

While the reproductive strategies demonstrated by *C. laevis* share similar characteristics to related species, the unusual combination of features explored here raise interesting questions about their adaptive advantage and could explain why they may be found at high densities in localized areas of the intertidal and the subtidal zones of eastern Canada (J-F Hamel, personal observation). This study on *C. laevis* provides information on an understudied and, in some ways, unique species of apodid. By characterizing patterns of gametogenesis, spawning, and development, we are able to assess how this species aligns with others of the same order and emphasize the ecological and evolutionary value of this holothuroid as a new model organism.

## Figures and Tables

**Table 1:** Developmental markers and size of embryos and juveniles of *Chiridota laevis* recorded throughout the first 2.5 y of growth.

Developmental Marker	Time	Size (mm)
Fertilized ootids	0	0.3
Cells 1–4	<12 h	0.3
Cells 8–32	24 h	0.3
Cells 32–128	48 h	0.3
Degradation of unfertilized ootids	4 d	0.3
Blastopore development	7 d	0.3
Gastrulation (appearance of the archenteron)	8–40 d	0.3
First appearance of pentaradial symmetry	43 d	0.3
Appearance of the first 5 tentacles	45 d	0.3
Presence of the calcareous ring	45 d	0.3
Hatching/pentactula	49 d	0.3
Burrowing	49 d	0.3
Intestine development	50–68 d	0.3
First tentacle ramification	68 d	0.3
First feeding	75 d	0.74
First sign of the Polian vesicle	75 d	0.74
First sign of posterior ossicle development (1st aggregate)	6 mo	1.52–2.87
Second tentacle ramification (4 digits)	6 mo	1.52–2.87
Development of 6th tentacle	6 mo	1.52–2.87
First sign of anterior ossicle (2nd aggregate)	6 mo	1.52–2.87
Development of ossicle aggregates 4–7	12 mo	2.35–3.60
Development of tentacles 7–8	12 mo	2.35–3.60
Development of ossicle aggregates 5–8	14 mo	2.35–4.1
Development of tentacles 9–10	1.5 y	2.35–4.1
Development of ossicle aggregates 5–8	1.5 y	5.80–6.23
Third tentacle ramification (6 digits)	1.5 y	5.80–6.23
First sign of gonopore (or pore canal)	1.5 y	5.80–6.23
Development of ossicle aggregates 8–16	2 y	6.23–10.10
Development of ossicle aggregates 16–21	2.5 y	12.21–20.10
Development of tentacles 10–12	2.5 y	12.21–20.10
Fourth tentacle ramification (8 digits)	2.5 y	12.21–20.10

## Data Availability

All datasets generated for this study are available on request from the corresponding author.

## Supplementary Materials

The following supporting information can be downloaded at: https://www.mdpi.com/article/10.3390/biology14111471/s1, Table S1: Glossary of related terms, Video S1: Hermaphroditic gonad.

## References

[1] Pierrat J., Bédier A., Eeckhaut I., Magalon H., Frouin P. (2022). Sophistication in a seemingly simple creature: A review of wild holothurian nutrition in marine ecosystems. Biol. Rev..

[2] Mercier A., Gebruk A., Kremenetskaia A., Hamel J.-F., Mercier A., Hamel J.-F., Suhrbier A.D., Pearce C.M. (2024). An overview of taxonomic and morphological diversity in sea cucumbers (Holothuroidea: Echinodermata). The World of Sea Cucumbers.

[3] Miller A.K., Kerr A.M., Paulay G., Reich M., Wilson N.G., Carvajal J.I., Rouse G.W. (2017). Molecular phylogeny of extant Holothuroidea (Echinodermata). Mol. Phylogenet. Evol..

[4] Ayranci K., Dashtgard S.E. (2013). Infaunal holothurian distributions and their traces in the Fraser River delta front and prodelta, British Columbia, Canada. Palaeogeogr. Palaeoclimatol. Palaeoecol..

[5] Reich M., Wiese F. (2010). Apodid sea cucumbers (Echinodermata: Holothuroidea) from the Upper Turonian of the Isle of Wolin, NW Poland. Cretac. Res..

[6] Reich M. (2015). Different pathways in early evolution of the holothurian calcareous ring. Prog. Echino. Palaeo..

[7] Pawson D.L., Fell H.B. (1965). A revised classification of the dendrochirote holothurians. Breviora.

[8] Zhang L., He J., Tan P., Gong Z., Qian S., Miao Y., Zhang H.-Y., Tu G., Chen Q., Zhong Q. (2022). The genome of an apodid holothuroid (*Chiridota heheva*) provides insights into its adaptation to a deep-sea reducing environment. Commun. Biol..

[9] Yamana Y., Tanaka H. (2017). A new species of *Chiridota* (Echinodermata: Holothuroidea: Apodida: Chiridotidae) from Japan, and first record of *C. rigida* from Japan. Zootaxa.

[10] O’Loughlin P., VandenSpiegel D. (2010). A revision of Antarctic and some Indo-Pacific apodid sea cucumbers (Echinodermata: Holothuroidea: Apodida). Mem. Mus. Vic..

[11] Woo S.P., Tan S.H., Nooraini I., Jaya-Ram A., Fujita T. (2021). A review on knowledge and research of interstitial sea cucumber. Paleontol. J..

[12] Eckelbarger K.J., Riser N.W. (2013). Derived sperm morphology in the interstitial sea cucumber *Rhabdomolgus ruber*, with observations on oogenesis and spawning behavior. Invertebr. Biol..

[13] Mercier A., Hamel J.-F. (2009). Endogenous and exogenous control of gametogenesis and spawning in echinoderms. Adv. Mar. Biol..

[14] Chao S.M., Chen C.P., Alexander P.S. (1995). Reproductive cycles of tropical sea cucumbers (Echinodermata: Holothuroidea) in southern Taiwan. Mar. Biol..

[15] Kubota T., Tomari M. (1998). Reproduction in the apodid sea sucumber *Polycheira rufescens*: Semilunar spawning rhythm and sex change. J. Mar. Biol. Assoc. UK.

[16] Smiley S., McEuen F.S., Chaffee C., Krishnan S., Giese A.C., Pearse J.S., Pearse V.B. (1991). Echinodermata: Holothuroidea. Reproduction of Marine Invertebrates.

[17] Sewell M.A., Chia F.S. (1994). Reproduction of the intraovarian brooding apodid *Leptosynapta clarki* (Echinodermata: Holothuroidea) in British Columbia. Mar. Biol..

[18] Sewell M.A. (1994). Birth, recruitment and juvenile growth in the intraovarian brooding sea cucumber *Leptosynapta clarki*. Mar. Ecol. Prog. Ser..

[19] Arakaki S., Yamahira K., Tokeshi M. (1999). Sex change and spatial distribution pattern in an intertidal holothurian *Polycheira rufescens* in the reproductive season. Res. Pop. Ecol..

[20] Kubota T. (2000). Reproduction in the apodid sea cucumber *Patinapta ooplax*: Semilunar spawning cycle and sex change. Zool. Sci..

[21] Sewell M.A. (1994). Small size, brooding, and protandry in the apodid sea cucumber *Leptosynapta clarki*. Biol. Bull..

[22] Green J.D. (1978). The annual reproductive cycle of an apodous holothurian, *Leptosynapta tenuis*: A bimodal breeding season. Biol. Bull..

[23] Ezhova O.V., Ershova N.A., Malakhov V.V. (2017). Microscopic anatomy of the axial complex and associated structures in the sea cucumber *Chiridota laevis* Fabricius, 1780 (Echinodermata, Holothuroidea). Zoomorph.

[24] O’Loughlin P., VandenSpiegel D. (2007). New apodid species from southern Australia (Echinodermata: Holothuroidea: Apodida). Mem. Mus. Vic..

[25] Gotto D.M., Gotto R.V. (1972). *Labidoplax media* Oestergren: A sea-cucumber new to British and Irish waters, with observational notes. Ir. Nat. J..

[26] Atwood D.G. (1973). Ultrastructure of the gonadal wall of the sea cucumber *Leptosynapta clarki* (Echinodermata: Holothuroidea). Z. Zellforsch. Mikrosk. Anat..

[27] Menker D. (1970). Lebenszyklus, jugendentwicklung und geschlechtsorgane von *Rhabdomolgus ruber* (Holothuroidea: Apoda). Mar. Biol..

[28] Sewell M.A. (1996). Mortality of pentactulae during intraovarian brooding in the apodid sea cucumber *Leptosynapta clarki*. Biol. Bull..

[29] Frick J. (1995). Reproductive Biology, Gonadal Microanatomy, and Parental-Embryonic Interactions in the Viviparious Holothurian Echinoderm *Synaptula hydriformis*. Ph.D. Thesis.

[30] Hamel J.-F., Himmelman J.H., Dufresne L. (1993). Gametogenesis and spawning of the sea cucumber *Psolus fabricii* (Duben and Koren). Biol. Bull..

[31] Frick J.E., Ruppert E.E., Wourms J.P. (1996). Morphology of the ovotestis of *Synaptula hydriformis* (Holothuroidea, Apoda): An evolutionary model of oogenesis and the origin of egg polarity in echinoderms. Invertebr. Biol..

[32] Jobson S., Hamel J.-F., Mercier A. (2024). A rare case of intra-ovarian oocyte maturation. Zygote.

[33] Burke R.D., Bouland C. (1989). Pigmented follicle cells and the maturation of oocytes in the sand dollar *Dendraster excentricus*. Dev. Growth Differ..

[34] Smiley S. (1990). A review of echinoderm oogenesis. Electron. Microsc. Tech..

[35] Clark H.L. (1910). The development of an apodous holothurian (*Chiridota rotifera*). J. Exp. Zool..

[36] Frick J.E. (1998). Evidence of matrotrophy in the viviparous holothuroid echinoderm *Synaptula hydriformis*. Invertebr. Biol..

[37] Byrne M., Sewell M.A., Selvakumaraswamy P., Prowse T.A.A. (2006). The larval apical organ in the holothuroid *Chiridota gigas* (Apodida): Inferences on evolution of the ambulacrarian larval nervous system. Biol. Bull..

[38] Sewell M., Prowse T., Selvakumaraswamy P., Byrne M. (2024). Larval development in the apodid sea cucumber *Chiridota gigas*, with a focus on coelom development and the serotonergic nervous system during metamorphosis. Invertebr. Biol..

[39] Lawrence J., Herrera J. (2000). Stress and deviant reproduction in echinoderms. Zool. Stud..

[40] Sewell M.A., Koss R.O.N., Turner A., Chia F.-S. (2006). Evidence for matrotrophy in the viviparous sea cucumber *Leptosynapta clarki*: A role for the genital haemal sinus?. Invertebr. Reprod. Dev..

[41] Sewell M.A., Chia F.-S., Thandar A.S. (1994). A redescription of *Leptosynapta clarki* Heding (Echinodermata: Holothuroidea) from the northeast Pacific, with notes on changes in spicule form and size with age. Can. J. Zool..

[42] Everingham J.W. (1961). The Intra-Ovarian Embryology of *Leptosynapta clarki*. Master’s Thesis.

[43] Turner R. (1973). Release mechanisms for gametes and juveniles of the hermaphroditic coelom-brooder *Synaptula hydriformis* (Echinodermata: Holothuroidea). Am. Zoool..

[44] Curtis M.D., Turner R.L. (2019). Development and morphology of ciliary urns in the sea cucumber *Synaptula hydriformis* (Echinodermata: Holothuroidea). Invertebr. Biol..

[45] Clark H.L. (1907). The Apodous Holothurians: A Monograph of the Synaptidæ and Molpadiidæ.

[46] Edwards C.L. (1907). The holothurians of the North Pacific coast of North America collected by the Albatross in 1903. Proc. US Natl. Mus..

[47] Kerr A.M., Kim J. (2001). Phylogeny of Holothuroidea (Echinodermata) inferred from morphology. Zool. J. Linn. Soc..

[48] Lacey K.M.J., McCormack G.P., Keegan B.F., Powell R. (2005). Phylogenetic relationships within the class holothuroidea, inferred from 18S rRNA gene data. Mar. Biol..

[49] Smirnov A.V. (2014). Sea cucumbers symmetry (Echinodermata: Holothuroidea). Paleontol. J..

[50] Gao F., Yang H., Yang H., Hamel J.-F., Mercier A. (2015). Anatomy. The Sea Cucumber Apostichopus japonicus: History, Biology and Aquaculture.

[51] Ferguson J.C. (1996). Madreporite function and fluid volume relationships in sea urchins. Biol. Bull..

[52] Ferguson J.C. (1992). The function of the madreporite in body fluid volume maintenance by an intertidal starfish, *Pisaster ochraceus*. Biol. Bull..

[53] Jobson S., Penney H.D., Hamel J.-F., Mercier A. (2020). Split personality. Front. Ecol. Environ..

[54] Sewell M.A., Hamel J.-F., Mercier A., Mercier A., Hamel J.-F., Suhrbier A.D., Pearce C.M. (2024). Morphological diversity, development, and biology of sea cucumber larvae. The World of Sea Cucumbers.

[55] Sun J., Hamel J.-F., Gianasi B.L., Mercier A. (2019). Age determination in echinoderms: First evidence of annual growth rings in holothuroids. Proc. R. Soc. B.

[56] Hamel J.-F., Sun Z., Mercier A. (2010). Influence of size and seasonal factors on the growth of the deep-sea coral *Flabellum alabastrum* in mesocosm. Coral Reefs.

[57] Morgan A. (2012). Use of a growth model to estimate size at age in the temperate sea cucumber *Australostichopus mollis*. SPC Beche-de-mer Inf. Bull..

[58] Watanabe S., Sumbing J., Lebata J. (2014). Growth pattern of the tropical sea cucumber, *Holothuria scabra*, under captivity. JARQ.

[59] Ghiselin M.T. (1969). The evolution of hermaphroditism among animals. Q. Rev. Biol..

[60] Brockington S., Clarke A. (2001). The relative influence of temperature and food on the metabolism of a marine invertebrate. J. Exp. Mar. Biol. Ecol..

[61] Ru X., Zhang L., Liu S., Sun J., Yang H. (2018). Energy budget adjustment of sea cucumber *Apostichopus japonicus* during breeding period. Aquac. Res..

[62] Dobson W.E., Stancyk S.E., Clements L.A., Showman R.M. (1991). Nutrient translocation during early disc regeneration in the brittlestar *Microphiopholis gracillima* (Stimpson) (Echinodermata: Ophiuroidea). Biol. Bull..

[63] Hamel J.-F., Mercier A. (1996). Gonad morphology and gametogenesis of the sea cucumber *Cucumaria frondosa*. Beche-de-mer Info Bull..

[64] Shanks A.L., Rasmuson L.K., Valley J.R., Jarvis M.A., Salant C., Sutherland D.A., Lamont E.I., Hainey M.A.H., Emlet R.B. (2020). Marine heat waves, climate change, and failed spawning by coastal invertebrates. Limnol. Oceanogr..

[65] Mercier A., Hamel J.-F. (2008). Depth-related shift in life history strategies of a brooding and broadcasting deep-sea asteroid. Mar. Biol..

[66] McEuen F.S. (1988). Spawning behaviors of northeast Pacific sea cucumbers (Holothuroidea: Echinodermata). Mar. Biol..

[67] Rogers A., Hamel J.-F., Quetzal J., Mercier A. (2021). Unique reproductive biology of the broadcasting sea cucumber *Holothuria floridana*: Facultative recruitment on adults inside nursery grounds. Invertebr. Reprod. Dev..

[68] MacIntosh H., de Nys R., Whalan S. (2014). Contrasting life histories in shipworms: Growth, reproductive development and fecundity. J. Exp. Mar. Biol. Ecol..

[69] Pawson D.L., Gage J.D., Belyaev G.M., Mironov A.N., Smirnov A.V. (2003). The deep sea synaptid *Protankyra brychia* (Echinodermata: Holothuroidea) and its near-surface dwelling planktotrophic larva, *Auricularia nudibranchiata*. Sarsia.

[70] Peters-Didier J., Sewell M.A. (2017). Maternal investment and nutrient utilization during early larval development of the sea cucumber *Australostichopus mollis*. Mar. Biol..

[71] Semon R. (1888). Die Entwickelung der *Synapta digitata* und ihre bedeutung für die phylogenie der Echinodermen. Jenaische Z. Naturwissenschaft.

[72] Engstrom N.A. (1980). Development, natural history and interstitial habits of the apodous holothurian *Chiridota rotifera* (Pourtales, 1851) (Echinodermata: Holothuroidea). Brenesia. San Jose.

