# Supplementary material for: Reproductive Biology and Early Life History of the Apodid Sea Cucumber Chiridota laevis
Source: Biology (Basel). 2025 Oct 22;14(11):1471. doi: 10.3390/biology14111471 (PMC12649911; doi:10.3390/biology14111471)
Supplement: Supplementary file 1 [file biology-14-01471-s001.zip › biology-3934768-supplementary.pdf]

biology MDPI

Supplementary Information

Table S1: Glossary of related terms.

Term	Definition
**Dioecy**	Dioecy, also called gonochorism is when individuals demonstrate either male or female gametes but not both.
**Follicular cells**	Follicular cells help to modulate the growth and maturation of oocytes through hormone release
**Germinal vesical (break down)**	The germinal vesicle (GV) contains the genetic information of a developing oocyte acting as the nucleus. The breakdown of the germinal vesicle (GVBD) occurs immediately prior to the first meiotic division with the dissolution of the GV freeing genetic material (chromatin) to mix with the cytoplasm of the oocyte prior to meiotic division.
**Gonad growth**	A pre-spawning stage of gametogenesis where the gametes are growing from immature to mature
**Gonad recovery**	A post-spawning stage of gametogenesis where any residual, unspawned gametes are broken down via cellular digestion (phagocytosis) and removed from the gonad.
**Hermaphrodism**	Hermaphrodism is when individuals demonstrate both male and female gametes. The expression of these gametes can occur at the same time (simultaneous hermaphrodism) or in sequence (sequential/reciprocal hermaphrodism)
**Lecithotrophy**	The nutrient mode where developing young receive all nutrients from the egg yolk.
**Matrotrophy**	The nutrient mode where the nutrient requirements of developing young are supplement by the mother (e.g., some introvarian larvae of apodids will feed on the coelomic matter held with the brooding/general body cavity of the mother).
**Oocyte**	An immature female gamete that will undergo meiotic division to create an ootid.
**Oogonim**	An immature female gamete (2n) that divides via mitosis to create oocytes
**Ootid**	A female gamete (1n) that has gone through both meiotic divisions but is still encased in the follicle
**Oviparous**	Reproduction by egg (e.g., broadcast spawners)
**Ovum**	A mature female gamete has escaped from the follicle and is ready to be fertilized
**Planktotrophy**	The nutrient mode where developing young feed on particulate matter.
**Polar bodies**	Polar bodies are a haploid by-product of meiotic division occurring in both meiosis I and II. These cells are much smaller than the parent cell and quickly degrade following formation.
**Primary oocyte**	An oocyte that has not undergone any meiotic division (2n)
**Protandry**	Protandry is when an individual switches between producing male and female gametes. Traditionally this reproductive trait has been assigned to species that only from male to female but more recent literature has used this term to describe a switch in gamete production from either sex.

**Realized fecundity**	The number of ootids successful spawned by one individual in a single spawning season.
**Reproductive mode**	The process by which an organism reproduces. Broadly, this could be split into sexual vs. asexual reproduction. However within each of those overarching categories, there are many other modes (e.g., viviparous, oviparous etc).
**Secondary oocyte**	An oocyte that has undergone the first meiotic division (1n)
**Vitellogenic oocyte**	Oocytes where the yolk protein (vitellogenin) has been deposited into the egg
**Viviparous**	The reproductive mode by which an individual grows young inside its own body (e.g., brooders)
